# The Symphony of Team Flow in Virtual Teams. Using Artificial Intelligence for Its Recognition and Promotion

**DOI:** 10.3389/fpsyg.2021.697093

**Published:** 2021-09-08

**Authors:** Corinna Peifer, Anita Pollak, Olaf Flak, Adrian Pyszka, Muhammad Adeel Nisar, Muhammad Tausif Irshad, Marcin Grzegorzek, Bastian Kordyaka, Barbara Kożusznik

**Affiliations:** ^1^Department of Psychology, University of Lübeck, Lübeck, Germany; ^2^Department of Social Science, Institute of Psychology, University of Silesia in Katowice, Katowice, Poland; ^3^University of Silesia in Katowice, Katowice, Poland; ^4^Department of Human Resource Management, College of Management, University of Economics in Katowice, Katowice, Poland; ^5^Institute of Medical Informatics, University of Lübeck, Lübeck, Germany

**Keywords:** team flow, team effectiveness, virtual teams, machine learning, collective communication

## Abstract

More and more teams are collaborating virtually across the globe, and the COVID-19 pandemic has further encouraged the dissemination of virtual teamwork. However, there are challenges for virtual teams – such as reduced informal communication – with implications for team effectiveness. Team flow is a concept with high potential for promoting team effectiveness, however its measurement and promotion are challenging. Traditional team flow measurements rely on self-report questionnaires that require interrupting the team process. Approaches in artificial intelligence, i.e., machine learning, offer methods to identify an algorithm based on behavioral and sensor data that is able to identify team flow and its dynamics over time without interrupting the process. Thus, in this article we present an approach to identify team flow in virtual teams, using machine learning methods. First of all, based on a literature review, we provide a model of team flow characteristics, composed of characteristics that are shared with individual flow and characteristics that are unique for team flow. It is argued that those characteristics that are unique for team flow are represented by the concept of collective communication. Based on that, we present physiological and behavioral correlates of team flow which are suitable – but not limited to – being assessed in virtual teams and which can be used as input data for a machine learning system to assess team flow in real time. Finally, we suggest interventions to support team flow that can be implemented in real time, in virtual environments and controlled by artificial intelligence. This article thus contributes to finding indicators and dynamics of team flow in virtual teams, to stimulate future research and to promote team effectiveness.

## Introduction

Advances in information and communication technology (ICT) provided the opportunity for virtual (team-) work and – due to globalization – more and more teams work together virtually over the globe (Jarvenpaa and Leidner, 1999; Raghuram et al., 2019). Organizations have adopted virtual teams for two main reasons. First, virtual teams are related to significant savings, such as reduced costs and time for traveling, and reduced meeting times. Second, virtual teams lead to higher flexibility, enabling organizations to cope with modern challenges stemming from globalization, competition, changing organizational structures, and increasing service demands (Purvanova, 2014). In addition, the COVID-19 pandemic has boosted the implementation of virtual team work, with many employees working from home using virtual tools to collaborate with their teammates (Feitosa and Salas, 2020).

However, formal and informal interaction is different in virtual teams, including communication among team members and team leadership. For example, reduced informal interaction in virtual teams leads to difficulties to build trust among virtual team members. Trust is however crucial when team members decide to ask each other for help, mutually provide feedback, and address issues and conflicts (Bell and Kozlowski, 2002; Jarman, 2005; Purvanova, 2014). These factors have significant effects on team effectiveness, which per definition includes performance measures as well as team members’ satisfaction with their working experience (Gilson et al., 2014; Pyszka, 2015a, b). Accordingly, coping with the particular challenges of virtual team work is essential for virtual teams.

A concept with a high potential for fostering team effectiveness is the concept of team flow (van den Hout et al., 2018). Team flow is a shared experience of flow, characterized by the pleasant feeling of absorption in an optimally challenging activity (Peifer and Engeser, 2021), and of optimal team-interaction during an interdependent task (van den Hout et al., 2018). Research on team flow is still scarce and particularly lacking for virtual teams. Furthermore, conditions of team flow will fluctuate during task completion and, thus, it is important to look for the dynamics of team-flow during the task. Team processes in general are dynamic phenomena but in current research we observe predominantly static treatment of team processes (Kozlowski and Chao, 2012).

However, assessing the dynamics of team flow is a challenge, as traditional measures of team flow are based on self-report questionnaires, which require an interruption of the team process. In order to study the dynamics of team flow during task completion, we thus need to identify a continuous, interruption-free team flow-indicator. Such an indicator can likely be found based on behavioral and sensor data. The evolutions in artificial intelligence and wearable sensor technology made it possible to collect physiological and sensor data and to predict emotional states. In this article, we will thus present an approach to measure team flow in virtual teams using machine learning methods. Based on a literature review, we will present physiological and behavioral characteristics of team flow. We will derive indicators which are suitable for machine learning in order to recognize them in real time. Finally, we will suggest interventions to foster team flow that can be implemented in real time, in virtual environments and controlled by artificial intelligence.

### Team Effectiveness and Trust in the Context of Virtual Teams

A team can be defined as “a small number of people with complementary skills who are committed to a common purpose, set of performance goals, and approach for which they hold themselves mutually accountable” (Katzenbach and Smith, 1993, p. 112). In difference to working groups, the performance of teams exceeds the mere sum of individual performances (Katzenbach and Smith, 1993). Furthermore, teams should be understood as complex, multilevel systems that function over time, tasks, and contexts (Pyszka, 2015b). The analysis of existing team effectiveness models shows a large variety of approaches and factors influencing team effectiveness. Input-Process-Output (IPO) models make predictions about the conditions and processes that lead to increased team effectiveness. One of the most popular IPO models has been developed by Hackman (1983) and he subdivides effectiveness into the components: task performance, ability to cooperate in the future, creativity, and satisfaction of team members. In recent years, research investigated team effectiveness in face-to-face compared to virtual teams. It was found that teams using computer mediated communication systems (CMCS) communicate less effectively in many circumstances than teams meeting face-to-face (Warkentin et al., 1997). A review of the literature indicates that the conditions that impact the effectiveness of virtual teams are still ambiguous (Ebrahim et al., 2009; Hertel et al., 2005; Purvanova, 2014). According to Olson and Olson (2006), the effectiveness of virtual teams is exposed to many challenges, including: the nature of work, the common ground of the team members, the competitive/cooperative culture, the level of technology competence of the team members, and the level of technical infrastructure in which the work resides. The most commonly reported challenge in virtual team work is that virtual communication is not an adequate substitute for face-to-face communication (de Guinea et al., 2012) which might lead to a lack of trust in colleagues (Baskerville and Nandhakumar, 2007).

There are different facets of trust, which become visible in at least two different definitions of the phenomenon. Accordingly, trust can be defined as “one’s expectations, assumptions, or beliefs about the likelihood that another’s future actions will be beneficial, favorable, or at least not detrimental to one’s interests” (Robinson, 1996, p. 576). Another definition understands trust as a party’s “willingness to be vulnerable to the action of another party based on the expectation that the other will perform a particular action important to the trustor, irrespective of the ability to monitor or control that other party” (Mayer et al., 1995, p. 712; see also Das and Teng, 1998; Man and Roijakkers, 2009).

Due to the lack of informal interaction, lack of knowledge about what others are doing, trust is difficult to build in virtual teams (Olson and Olson, 2006; Breuer et al., 2016). Trust determines whether team members ask each other for help, share feedback, and discuss issues and conflicts (Breuer et al., 2016; de Jong et al., 2016). Therefore, trust has a significant effect on team effectiveness (de Jong et al., 2016). This represents an entirely new paradigm of communication that is needed in *virtual* teams, that must be learned, with little means of social control, with new tools and techniques of social interaction which need to foster familiarity and proficiency (Warkentin et al., 1997).

### Effects of Flow on Trust and Team Effectiveness in Virtual Teams

A concept with a high potential for fostering trust and team effectiveness is the concept of team flow (van den Hout et al., 2018). Previous research already indicated that the concept of flow can be a meaningful antecedent of trust in virtual settings (Bilgihan et al., 2015), whereby the presence of flow increased the perception of trust. A potential explanation is that positive emotions resulting from the experience of flow contribute to building an atmosphere of benevolence in which team members feel good and rightly. Simultaneously, shared positive experiences foster trust in the team’s achievement as well as reciprocal stimulation and inspiration. Maintaining such beneficial conditions in the work team over time conveys a sense of safety and stability, as well as dependability and trustworthiness – thereby fostering different facets of trust, i.e., positive expectations toward team members’ future actions and low need to control others.

A broad empirical evidence exists for the links between flow and efficiency (for an overview see Peifer and Wolters, 2021). Those links have been confirmed for different efficiency variables, such as increased wellbeing (e.g., Peifer et al., 2020b), work satisfaction (Maeran and Cangiano, 2013), qualitative and quantitative performance (Peifer and Zipp, 2019), in- and extra-role performance (Demerouti, 2006), learning outcomes (Engeser and Rheinberg, 2008), service quality (Kuo and Ho, 2010), and creativity (Zubair and Kamal, 2015). Also, in teams performing complex planning tasks, team flow was found to be positively related to team performance (Heyne et al., 2011). Similarly, a study investigating student teams performing a project management task found that the flow of team members was associated with team performance (Aubé et al., 2014). Such positive associations of team flow with team performance were also found in a video game experiment (Keith et al., 2014) as well as in the work context (van den Hout et al., 2019). In a longitudinal study, students were asked to compose a piece of music and their flow experience during the process was positively related to creativity of the team product as assessed by an expert jury (MacDonald et al., 2006), which provides evidence also for long-term effects of flow on team effectivity.

### Components of Team Flow

Team flow needs to be distinguished from individual flow. *Individual flow* is a pleasant experience of being fully absorbed in an optimally challenging task (Csikszentmihalyi, 1975, 1990). Its core characteristics are a high degree of absorption with the task, a perceived demand-skill balance, and enjoyment (Peifer and Engeser, 2021). Building upon the definition of individual flow, team flow has been defined as “a shared experience of flow derived from an optimized team dynamic during the execution of interdependent personal tasks” (van den Hout et al., 2018, p. 400). As a shared experience of flow, team flow shares the characteristics of individual flow, i.e., a high degree of absorption with the task, a perceived demand-skill balance, and enjoyment (Pels et al., 2018; van den Hout et al., 2018; Peifer and Engeser, 2021); but entails additional, team flow-specific characteristics that reflect the social nature of the phenomenon (Pels et al., 2018).

The literature on flow in social situations is yet quite scarce and within this literature, the approaches to the concept vary from individual flow in social contexts to interdependent flow in dyads or teams (Walker, 2021). Terms that can be found in literature are e.g., social flow (Walker, 2010), collective flow (Quinn, 2003, 2005; Šimleša, 2018), group flow (Sawyer, 2003, 2006, 2008), and team flow (van den Hout et al., 2018), to name the most common ones. In the following, when talking about *team flow*, we will refer to those social flow phenomena, which are described as *a shared social experience during a group’s interdependent interaction.*

Sawyer (2003) was one of the firsts who proposed a concept of group flow. He emphasized the interdependence of the group as a particular characteristic of group flow as compared to individual flow. With his emphasis on interdependence, Sawyer’s understanding of group flow aligns with our understanding of team flow. Sawyer proposed a clear differentiation between group flow and individual flow, claiming that the group can be in flow when the members are not experiencing individual flow and that members can be in individual flow while the group is not in flow (Sawyer, 2008). According to this claim, characteristics of group flow may in part be different from characteristics of individual flow. Based on a qualitative approach (although the details of this study were not published), Sawyer (2008) discussed 10 conditions of group flow: (1) the group’s goal (clear vs. open depending on the task), (2) close listening to one another, (3) complete concentration to the group task, (4) being in control of their actions and environment, (5) the ability of group members to merge their egos with the group mind, (6) equal participation of group members, (7) familiarity with group members performance styles, a shared understanding of the group’s goals and conventions, and shared tacit knowledge, (8) constant and spontaneous communication, (9) moving the process forward, and (10) the potential for failure.

In his approach, Quinn (2003, 2005) defines “collective flow” as the experience of “moving together toward shared or complementary goals, adjusting in real time to each other’s expectations, needs, and contributions, and learning how others work and how to interact effectively along the way” (p. 637). With this definition, he points to the dynamic nature of team flow in team processes, in which team members need to react to each other. Similar to Sawyer (2006), Quinn differentiates between individual flow and team flow (what he calls collective flow) and proposes additional distinct conditions for team flow: (1) the coordination of activities, (2) a collective goal that structures the joint activity, and (3) comparable skill levels. And also, Walker (2010, 2021) differentiates individual flow and team flow (what he calls “interactive social flow”), and as further conditions of team flow he suggests: (1) agreement on goals, procedures, roles, and patterns of interpersonal relations and (2) uniformly high competency of team members (Walker, 2010). In a similar vein, van den Hout et al. (2018) propose that “team flow has similar conditions as individual flow, but teams are subject to additional considerations, specifically team communication, information sharing, and team member perceptions of teammate performance and effort” (p. 400). Based on this, and on previous literature on social flow, van den Hout et al. (2018) proposed a team flow model, with the following antecedents: (1) collective ambition, (2) common goal, (3) aligned personal goals, (4) high skill integration, (5) open communication (6) safety, and (7) mutual commitment. In an empirical study using a cross-sectional approach, van den Hout et al. (2019) found evidence for their proposed team flow conditions. In their scoping review on group flow, Pels et al. (2018) also distinguished between individual aspects of group flow and collective aspects of group flow. Individual aspects identified in the literature (compare Pels et al., 2018, table 1, p. 6ff) were the individual experience of flow, as well as the flow characteristics enjoyment, demand-skill balance and absorption (including feeling one with the group). These individual aspects are in line with the core characteristics of flow according to Peifer and Engeser (2021). In their summary of empirical findings on group flow, they further list “aspects of competence (e.g., knowing others’ skills; Kaye and Bryce, 2012), interaction (e.g., effective communication; Kaye, 2016), and of positive relationships (e.g., trust within the group; Armstrong, 2008)” as antecedents of group flow. Other identified aspects within the definitions provided in the literature (compare Pels et al., 2018, table 1, p. 6ff) relate to the aspect of common goals such as “purposeful communication” (Duff et al., 2014), “concurrent engagement in a shared goal-oriented activity” (Hart and Di Blasi, 2015), or “common focus” (Kaye and Bryce, 2014). Also, reported aspects of group flow according to Pels et al. (2018, table 1, p. 6ff) are that of interactional synchrony (Zumeta et al., 2016) and social contagion (Bakker et al., 2011; Aubé et al., 2014).

Despite the variety of the proposed terms, characteristics and conditions of team flow, the characteristics and conditions of team flow show commonalities on a level of content. As can be seen in Table 1, the conditions of team flow as described by the just referenced authors can be summarized into four major categories: (1) communication and feedback, (2) goal commitment, (3) equal participation, and (4) trust.

**TABLE 1:** Meta-categories of team flow-specific characteristics.

Meta-category	Sawyer (2003, 2008)	Quinn (2003, 2005)	Walker (2010)	van den Hout et al. (2018)	Pels et al. (2018)
Communication and feedback	Close listening to one another, constant and spontaneous communication	The coordination of activities	Agreement on goals, procedures, roles, and patterns of interpersonal relations	Open communication	Interaction, e.g., effective communication; fluent, positive interactions within the group (p. 18)
Shared goal commitment	A shared understanding of the group’s goal, complete concentration to the group task, the ability of group members to submerge their egos to the group mind, shared understanding of the group’s goals and conventions, and shared tacit knowledge	A collective goal that structures the joint activity	Agreement on goals, procedures, roles, and patterns of interpersonal relations	Collective ambition, common goal, aligned personal goals, and mutual commitment	Common goals, e.g., purposeful communication, concurrent engagement in a shared goal-oriented activity, or common focus
Equal participation	Equal participation of group members	Comparable skill levels	Uniformly high competency of team members	High skill integration	Interactional synchrony; contagion effect; a shared state of balance; a high collective competence
Trust in each other’s knowledge, skills, and attitudes	Being in control of their actions and environment, familiarity with group members performance styles, a shared understanding of the group’s goals and conventions, and shared tacit knowledge	Comparable skill levels	Uniformly high competency of team members	A shared belief that the team is safe; mutual trust as characterized by: (a) a willingness to be vulnerable, (b) mutual respect, (c) confidence in the working environment, and (d) team potency/efficacy (p. 410)	Positive relationships, trust within the group; aspects of competence, knowing each other’s skills

*Some of the components are included in more than one category.*

Importantly though, we need to distinguish conditions from characteristics. Also for individual flow, there has been a long discussion about which of the flow characteristics are conditions, core components or outcomes (see e.g., Landhäußer and Keller, 2012). For some core components, such as the challenge-skill balance (or: demand-skill balance), some authors argue it is a condition, others count it as a component (Landhäußer and Keller, 2012). In the meantime, there is some agreement in the literature that the objective presence of a demand-skill balance is a condition of flow, while the perceived demand-skill balance is defined as a component (Peifer and Engeser, 2021). As the just described team flow conditions are unique for team flow as compared to individual flow, we argue that the perception of their presence can be regarded as a component of team flow.

Taken together, team flow is composed of those characteristics that are shared with individual flow combined with characteristics that are unique for team flow. The resulting components are listed in Table 2.

**TABLE 2:** Components of team flow.

Component	Shared between individual and team flow	Unique for team flow
Absorption	X	
Perceived demand-skill balance	X	
Enjoyment	X	
Communication and feedback		X
Shared goal commitment		X
Equal participation		X
Trust in each other’s knowledge, skills, and attitudes		X

While the literature on flow in social contexts (and on team flow specifically) is scarce, literature on team flow in virtual contexts barely exists, although virtual teams are increasingly important in today’s workplaces. Compared to real-world settings, we argue that achieving the outlined team flow characteristics: (1) communication and feedback, (2) shared goal commitment, (3) equal participation, and (4) trust are particularly challenging in virtual contexts, due to the lack of informal communication. Accordingly, reaching team flow in virtual teams should be more difficult than in face-to-face contexts. Even more so it is necessary to identify indicators that can measure team flow in virtual teams in order to find and evaluate approaches to promote team flow in virtual teams. To find such indicators, we should measure the presence of the characteristics of team flow in real time during the team process. A concept, which could help finding such indicators as it is largely overlapping with the specific team flow conditions, is the concept of collective communication.

### Collective Communication

According to Watzlawick and Beavin (1967), all behavior in the presence of another person is communicative (with presence going beyond physical presence). Thereby communication is more than verbal productions but includes all behavior in the social context (Watzlawick and Beavin, 1967). Communication is further based on individual characteristics like openness, and it can for example be direct and indirect, verbal and non-verbal, and as such it can be measured by different indicators like communication style or listening ability, and many others (Hargie and Tourish, 2000).

The term “*collective communication*” is a particular group-related communication style, which refers to a group’s (or team’s) behavior and does not necessarily correlate with individual communication (Kozusznik et al., 2018), although it may vary from the sum of individual factors (Woolley et al., 2010). Collective communication can be described as the connections among people, feedbacks, and interrelations (Weick and Roberts, 1993). It refers to the bundle of messages from all group members given at the same time or otherwise in the form of feedback. Each message provides direct or indirect feedback to the team members. Non-verbal social feedback can be derived from smiles, attention, tone of voice, or other social cues. They usually represent spontaneous reactions without intentions of teaching or otherwise influencing. Besides, it can be found that it does not induce additional cognitive loads (Knox, 2012). Collective communication appears when group participants have equal chances to participate and communicate in the discussion (Woolley et al., 2010). It includes specific forms of communication that guide and prioritize activities within the team while maintaining all its members’ equality and well-being. This ensures a spontaneous and expressive response in a safe and comfortable environment for the individual, allowing for convenient speech without fear of losing one’s meaning (Kożusznik, 2005).

It was found that collective communication strengthens the team members’ process control and improves knowing each other and mutual understanding (Riedl and Woolley, 2017). Operationally, it allows to reduce the redundancy of statements, thoughts, and actions, eliminates delays, facilitates the use of participants’ knowledge, and provides people crucial information and opportunities to perform. Specific indicators of collective communication are the motivational drivers of participation in the communication. Existing research confirms that equal communication gives rise to an equal rhythm of communication that can predict group performance on a wide variety of tasks (Woolley et al., 2010). Interrupting others is correlated with domineering (Kożusznik, 2005; Woolley et al., 2010). When one team member interrupts the speaker, it causes a decrease in effectiveness and impairs the speaker’s well-being (Bouskila-Yam and Kluger, 2011). The temporal patterning of activities is an important aspect of team effectiveness (McGrath, 1984). Alternating interaction is an orderly process of verbal and non-verbal activities which help regulate the flow of conversation, enable turn-taking, and provide feedback. Additionally, rather than a randomly distributed communication pattern, there tend to be periods of high activity (bursts) of the group followed by periods of little activity that enhance team flow (Riedl and Woolley, 2017). Questioning is also related to the effectiveness of the group performance (Bouskila-Yam and Kluger, 2011), as it leads to increased mutual understanding, improved team coordination and more clarity in the work process.

## Complementing a Team Flow Measure by Means of Collective Communication

When comparing the characteristics of team flow with the concept of collective communication, their strong overlap becomes evident, as shown in Table 3.

**TABLE 3:** Overlaps between collective communication and team flow.

Team flow characteristic	Characteristic of collective communication
Communication and feedback	Stems from the connections among people, feedback, and interrelations; ensures a spontaneous and expressive response^1^
Goal commitment	Includes specific forms of communication that guide and prioritize activities within the team^2^
Equal participation	Appears when group participants have equal chances to participate and communicate in the discussion^3^
Trust in each other’s knowledge, skills, and attitudes	Takes place in a safe and comfortable environment for the individual, allowing for convenient speech without fear of losing one’s meaning^4^

*^1^Weick and Roberts, 1993;*

*^2^McGrath, 1984; van Dyne et al., 2003; Bies, 2009; Ross, 2014;*

*^3^Weick and Roberts, 1993; Kożusznik, 2005; Woolley et al., 2010;*

*^4^Hietanen et al., 1998; Bouskila-Yam and Kluger, 2011.*

Accordingly, the concept of collective communication represents the team flow characteristics and it can be used to operationalize team flow indicators. Indicators of collective communication have already been identified, which can now be used to complement a measure of team flow using machine learning.

Collective communication correlates with a set of behavioral markers: equal distribution of conversational turn-taking, the number of speaking turns, silence, voice volume, number of interruptions, facilitating listening, space offering, “I” and “We” and burstiness (Kożusznik, 2005; Kluger and Nir, 2009; Woolley et al., 2010). Also, measures like time of speaking can measure it, as well as the number of questions, speed of talking, and others (see Table 5).

**TABLE 4:**
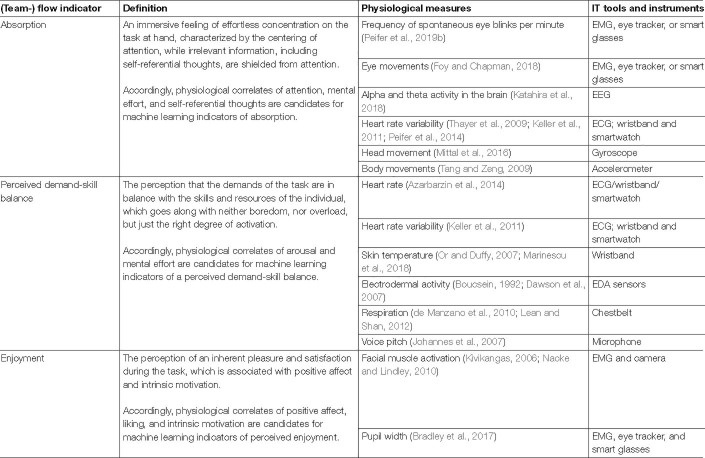
Physiological measures of flow experience applicable for machine learning as part of a team flow algorithm (compare Peifer and Engeser, 2021; Peifer and Tan, 2021).

**TABLE 5:**
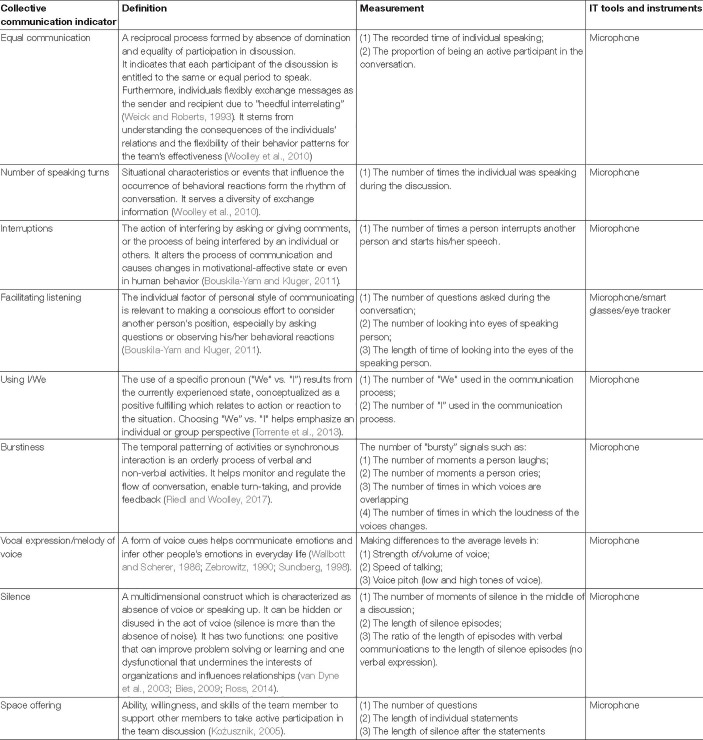
Definitions and measurement of collective communication enhancing team flow.

### Team Flow-Indicators Suitable for Machine Learning

As described above, team flow is composed of those characteristics that are shared with individual flow combined with characteristics that are unique for team flow (compare Table 2). Accordingly, a team flow measure should be operationalized based on all these characteristics.

For individual flow – i.e., for those team flow characteristics that are shared with individual flow – there are already elaborated concepts and studies on its physiological correlates (for an overview see Tozman and Peifer, 2016; Peifer and Tan, 2021) and their potential use for machine learning (Peifer et al., 2020a; Rissler et al., 2020). Studies show that individual flow experience is for example associated with heart rate variability (Peifer et al., 2014), electrodermal activity (de Manzano et al., 2010), respiration (de Manzano et al., 2010), blinking rate (Rau et al., 2017; Peifer et al., 2019a), or facial muscle activation (Kivikangas, 2006; de Manzano et al., 2010; Nacke and Lindley, 2010). Those indicators can be sorted according to the components of flow, i.e., if they relate to absorption, perceived demand-skill balance and/or enjoyment. In Table 4 we propose physiological measures that can be used to assess individual flow in real time and which are suitable for machine learning.

In order to complement the measurement of the just described team flow components, we need to include also indicators for those characteristics, that are unique for team flow, i.e., (1) communication and feedback, (2) shared goal commitment, (3) equal participation, and (4) trust. As discussed, this can be reached using the concept of collective communication, as behavioral measures of collective communication already exist (Table 5). An advantage of their measurement in virtual teams is, that they can be assessed using the video camera and the audio signal. Such indicators of collective communication are: (a) equal communication (b) number of speaking turns; (c) interruptions; (d) facilitating listening; (e) number of the use of We and I, (f) burstiness; (g) vocal expression/melody of team voice; (h) silence; and (i) space offering. The detailed definitions and measurement of those proposed indicators is described below and also presented in Table 5.

For measuring *equal communication* each subject can be assessed to gain information about “equal” vs. “unequal,” i.e., each group participant will be compared to the overall discussion time. Measurement of the *number of speaking turns* requires constant monitoring and counting changes in the course of discussion. Similarly, *interruptions* can be calculated during permanent monitoring with counting the number of times when a person interrupts another person and starts his/her speech. Collecting samples of *facilitating listening* can be achieved by recognizing the presence of particular actions such as questioning and focusing the current speaker. The *use of I/We* can be assessed by monitoring and counting the numbers of “We” and “I” in whole and separate parts of the communication process. For *burstiness* (or: liveliness), individual and continuous estimation will be used, and the measurement of each participant will be compared with results of other team members. *Vocal expression/the melody of the voice* can be measured via differences in average behavior, e.g., in speed of talking, voice pitch, and volume of voice. *Silence* requires continuous estimation and determination of its duration to compare the outcomes of different individuals.

### Using Machine Learning to Develop an Application-Based Algorithm for Team Flow Analysis

Machine learning approaches have been widely practiced in recent times by using data from video, motion, and physiological sensors for the recognition of physical and cognitive activities in the field of medical data science (Irshad et al., 2020; Nisar et al., 2020). Therefore, based on the indicators described in Tables 4, 5, it is possible to develop an application for team flow analysis in the form of an end-to-end machine learning-based algorithm that takes input from multiple sensors including wearable devices, cameras and microphones, and predict the cognitive states of the participants of virtual teams by analyzing not only their own physiological data but also their interaction and communication with other team members. In order to build such an application, it is necessary to answer several questions like how to handle the heteroscedasticity of different input signals (i.e., the variability of variance of errors of the input data), what will be the useful features (Amjad et al., 2021), which feature extraction and classifications techniques will be used? The artificial neural networks handle all these issues and are able to learn and model non-linear and complex relationships between input and output (Li et al., 2018, 2020). Therefore, end-to-end machine learning algorithms based on artificial neural networks can learn the whole path between the raw sensory data and the final outcomes, for example, the cognitive state and interactions of the participants of virtual teams. However, once the models are trained, it is hard to semantically understand the very complex processing between the input and the output. So, a deep neural network behaves like a black box that does not explain the reasons for particular outcomes. To explain and interpret the transformation between the input data vector and the output cognitive states, we must investigate the application of multi-task deep neural networks sharing some hidden layers and training others specifically for certain environmental and social constellations (e.g., team roles, time working as a team), certain tasks or task characteristics, and certain groups of people similar to each other in terms of their individual differences (e.g., personality, experience, age, gender, etc.). If we manage to explain the predictive decisions of our machine learning algorithms, we will generate new scientific findings in the area of cognitive state analysis. In other words, we will not only be able to implement our deep learning models as a black box, but we will also be able to describe the distinctive features found in the sensor data which are specific for team flow.

Another potential challenge is the situation that people could be too diverse in terms of individual differences or team roles (Driskell et al., 2017) – for one single machine learning approach analyzing their cognitive state and the interaction with their colleagues. It might come out that there are some different types (clusters) of people in the virtual teams having similar properties so that one machine learning configuration works better for one cluster, another for another. It would not be efficient to develop completely isolated machine learning systems for all clusters separately from each other. The machine learning system incorporating the parametric models for different clusters can be helpful to avoid the completely independent systems for each cluster type. When assigning concrete values to the parameters, the generic system can be converted into a concrete system for a certain cluster or team roles. Estimating these meta-parameters can be realized by a supervised machine learning approach.

To train the algorithm, subjective measures of team flow will be needed, potentially complemented by observer ratings. Even after an algorithm has been identified, such subjective measures should be used at least intermittently to complement objective data and to validate and improve the algorithm.

## Interventions to Foster Flow in Virtual Teams

A machine learning system that identifies team flow can help not only to measure, but also to promote team flow and its dynamics over time. This means, the machine learning system can be used as a decision support system, that can identify team processes as fostering or hindering for team flow and provide feedback if team processes deviate significantly from their optimal level. The level of support by the machine learning system can vary along a continuum of low to high support starting with information and feedback only, up to the proposal of suitable interventions (Peifer et al., 2020a). Also, the decision authority may range from full authority by the team members/team leader up to full authority by the system (Parasuraman et al., 2000), e.g., the system can mute speakers or send automated instructions on interventions or on how to proceed.

### What Could Real-Time Interventions Look Like?

The specific intervention depends on which team flow indicator deviates from its optimal level. In the case that indicators reflecting *absorption* deviate, the machine learning system could propose a pause or a meditation in order to regain energy and focus (Peifer and Tan, 2021). A deviation of the *perceived demand-skill balance* can be improved by re-defining individual goals, or social support. If *enjoyment* is lacking, the machine learning system could propose team interventions that foster positive emotions, such as providing compliments to each other, or the use of humor. A huge selection of potential interventions can be found in the field of positive psychology (Meyers et al., 2013).

If indicators reflecting *communication and feedback* deviate, e.g., the number of questions is small, or there is a high degree of silence, the machine learning system invites the discussant to ask questions. In instances of escaping (e.g., the listener is busy with her phone or computer), the system could remind the respective team member to join the team process.

If *shared goal commitment* is not given, e.g., as reflected by the use of “I” dominating the use of “We,” the machine learning system can invite participant(s) to use “We” instead of “I.” Using “We” strengthens the collective communication which resides in the connections between the units and the flexibility of their patterns of behavior (Weick and Roberts, 1993). The pronoun “I” represents an individual approach to measuring work engagement, the pronoun “We” represents the collective engagement conceptualized as a positive, fulfilling, work-related state shared with vigor and dedication (Schaufeli et al., 2002; Torrente et al., 2013).

If *equal participation* is not given – e.g., one person dominates and consumes too much time in the conversation – the system could inform this person (or the manager) and ask to shorten the time of speaking. When the system identifies that the number of speaking turns is small, it invites participants to make another round of conversation. In case of too many interruptions and talking into each other statement’s, the system asks to give some time and possibilities to others to let them talk.

If indicators of *trust* deviate, e.g., as reflected by the number of “bursty” signals (lough, cry, overlapping of the voices, and loudness of the voices), machine learning system could inform participants to stop or reduce these signals. Burstiness is related to levels of interpersonal synchrony or temporal coordination and influences trust (KoŻusznik and Polak, 2016) and effectiveness (McGrath, 1984). Also, e.g., if the machine learning system detects that the voice is too loud it can inform the participants and invite them to decrease the volume. If the machine learning system detects too low or too fast speed of talking it can ask the participant to regulate the speech. Similarly, the machine learning system could analyse the voice pitch and monotony and invite the participants to make the voice lower or more vivid. There is a correlation between group vocal expression and trust and well-being among its members (Hietanen et al., 1998). Also larger scale interventions to improve trust can be imagined (such as team building interventions), if a team continues to show signs of low trust among each other.

## Discussion

In this article we presented an approach to measuring team flow in virtual teams using machine learning methods. To provide a basis for the development of suitable data for the machine learning algorithm, we have disentangled the characteristics of team flow. We suggested that team flow is composed of characteristics that are shared with individual flow – i.e., absorption, perceived demand-skill balance and enjoyment –, and characteristics that are unique to team flow – i.e., communication and feedback, shared goal commitment, equal participation, and trust. Based on these characteristics and existing research on flow and collective communication, we have identified physiological and behavioral indicators that are suitable as machine learning input data. Furthermore, we have outlined how these data can be used in machine learning to develop an algorithm that assesses team flow in real time. Also, we have identified potential challenges in this endeavor. Finally, we have suggested how the real-time measurement of flow can result in interventions to improve team flow during the team process. In the following, we are discussing the underlying theoretical approach, as well as implications of our approach for research and practice.

### Underlying Theoretical Approach

To clarify the uniqueness of team flow, we chose a relational approach. The theory of relational models describes four fundamental forms of social relationships: communal sharing, authority ranking, equality matching, and cost benefit analysis (market pricing) (Fiske, 1991; Fiske and Haslam, 2005). Our assumptions are best supported by equality matching, which builds the basis for turn-taking, equal rights, even sharing, voting, and balanced reciprocity, as well as enabling people to return the same kind of thing they received (Haslam, 2004). Collective communication appears when group participants have equal chances to participate and communicate in the discussion (Woolley et al., 2010). It includes specific forms of communication that guide and prioritize activities within the team while maintaining all its members’ equality and well-being. This ensures a spontaneous and expressive response in a safe and comfortable environment for the individual, allowing for convenient speech without fear of losing one’s meaning (Kożusznik, 2005). The relational approach also allows us to capture the dynamics of team flow and, thus, to determine optimal conditions of the collective communication that enhances team flow in a virtual team.

However, also a motivational approach is relevant in the context of team flow, as the enjoyment of the task at hand, including the resulting intrinsic motivation, is a shared characteristic of flow and team flow. This also applies to the characteristics “absorption” and “perceived demand-skill balance,” which can be attributed to a cognitive approach. Accordingly, relational, motivational as well as cognitive indicators of team flow have been proposed as part of the suggested team flow measure and also as parts of potential interventions.

### Implications for Future Research

We proposed a machine learning system that employs multimodal sensory data to measure team flow in virtual teams. The heteroscedastic input data provided to the machine learning system not only cover physiological data of the members of a virtual team but also consider the vital key aspects of their communication with the co-members. The main objective of the machine learning system is indeed to detect the team flow using all sensory inputs, however, it will also be interesting in the future to see the impact of each kind of input signals in recognizing the team flow. The effectiveness of the machine learning system confides in detecting team flow with good accuracy as well as identifying the distinctive features of the input data that are specific for team flow. Explainable artificial intelligence administers the techniques which highlight the meaningful distinctive features in achieving the desired outcome of the system. A major advantage of an AI-based analysis of team flow is that team members do not need to be interrupted to measure team flow, which allows to assess the process with its fluctuations over time. Also, the automated analysis allows a more objective investigation of the context that can add to traditional self-report measures. In combination, self-report and machine learning data will allow to find a larger, more holistic model of team flow. Accordingly, the machine learning system can serve the function of a real laboratory and help to better understand the concept of team flow, the fluctuations of team flow over time and conditions that promote or hinder team flow.

A still unanswered question in flow research is the relationship between individual flow and team flow (van den Hout et al., 2018; Walker, 2021). Currently, there is no final agreement regarding the relationship of both flow forms of flow and their dynamics over time. The suggested machine learning system provides the opportunity to gain insights into their interplay by holistically relating flow and team flow indicators and sensor data. This will contribute substantial new information to the debate regarding the interplay between individual and social flow.

Also, the effects of different context factors can be studied in more detail. For example, it is well-documented that tasks have differential effects on the team process in terms of losses and gains, and, as a consequence, on team performance. Some tasks were for example found to facilitate social loafing, beyond them additive tasks (Kerr and Bruun, 1981), and easy tasks, while difficult tasks lead to increased performance (Jackson and Williams, 1985). Furthermore, group think was rather found in judgment tasks and less so in intellective tasks (McGrath, 1984). Using machine learning methods to detect team flow and team flow dynamics in different types of tasks could provide deeper information about which tasks are particularly flow-promoting or flow-hindering, about the mechanisms that are responsible, and also which characteristics of the flow experience are particularly affected (e.g., equal participation, goal commitment, perceived demand-skill balance, etc.). This helps us to achieve a better understanding of how to design tasks to achieve team flow and increase team efficiency. Also, this knowledge can be used to propose changes in the type of task in order to stimulate team flow in ongoing team processes. Future studies should thus systematically investigate task types and task characteristics by controlling for the type of task and/or systematically varying different tasks and task characteristics.

Another important domain that affects team processes relates to formal and informal social roles and relationships within the team (Belbin, 1981; Driskell et al., 2017). Teams mostly consist of a team manager and team members, with certain individual characteristics as well as formal and informal roles (Belbin, 1981). Team members including the team manager depend on each other, activated by managerial actions as a constellation of specific objectives, resources and processes (Sohmen, 2013). Also in virtual teams, challenges relate to difficulties in team leadership and the coordination of the team members’ activities (Pinjani and Palvia, 2013). Accordingly, the investigation of team constellations, in terms of team leadership style (e.g., teams managed by a leader vs. self-managed teams) and in terms of team roles and individual characteristics, are further relevant questions that should be systematically addressed in future research on team flow. Furthermore, there is likely an interplay between individual characteristics, formal and informal team roles, task characteristics, and the time working together as a team which is worth investigating. By means of the machine learning system, it will be possible to answer more complex questions about why some teams are more effective than others in the future. Corresponding findings can inform more advanced versions of the machine learning system using tools from user-centered design to differentiate between different groups of users within a team based on task characteristics, the team constellation, and the team members’ individual characteristics (Pyszka, 2015b).

### Implications for Practice

A machine learning system measuring team flow can be used in practice to identify team processes that are promoting or hindering team flow and to derive suitable interventions during the team process. This is even more relevant for practice, as team flow is highly related to team effectiveness – including team performance and team satisfaction (Peifer and Wolters, 2021). Such a machine learning system could complement existing online management tools (e.g., TransistorsHead.com) that are already used to record team members’ actions in virtual environments (Flak, 2013, 2019; Flak et al., 2017) and could be incorporated in a more holistic, artificial team management tool (Flak, 2020). It was found that declarations of team management processes based on memory are highly imprecise and subjective as compared to the objective parameters recorded by online management tools (Flak and Pyszka, 2013). Accordingly, the implementation of artificial team management tools has the potential to provide more objective feedback, more objective decision criteria and more suitable interventions to the team. Moreover, improving skills related to collective communication through the implementation of artificial team management could contribute to enhance relational links and information exchange in teams, as well as buffer the impact of personality and team role diversity.

The development and implementation of a machine learning system comes with substantial set-up costs. However, we follow Pyszka’s (2015a) argumentation and understand effectiveness in an evolutionary manner assuming that a change from economic efficiency assessment today toward the evaluation of the potential of solutions will enable even higher levels of effectiveness in the long-term.

Accordingly, the implementation of a machine learning system promises added value for organizations: the machine learning system can lead to higher levels of employee satisfaction, having a positive influence on productivity of an organization (Harter et al., 2002). The machine learning system could be developed even further and integrate additional sources of data (such as characteristics and preferences of employees) toward a holistic system of organizational team management. Due to its innovativeness and low dissemination the implementation of such a machine learning system, it promises competitive advantages over other competitors based on the opportunity for better teamwork, which can improve the process efficiency of an organization in an innovative and unique manner leading to advantageous market positions in the future (Dean, 2014).

## Conclusion

This article proposed an approach to measure and ultimately promote dynamic, not static, team functioning in virtual teams using machine learning methods. For this, the concept of team flow is a promising target state with high significance for team effectiveness. The concept of collective communication can provide suitable indicators of team flow specific characteristics, which can be used to complement a machine learning algorithm. Such an algorithm can then be used to not only identify, but also promote team flow, by providing feedback to the users and proposing interventions as part of an automated team management system.

## Data Availability Statement

The original contributions presented in the study are included in the article/supplementary material, further inquiries can be directed to the corresponding author/s.

## Author Contributions

CP, BKoż, APo, OF, APy, MN, MI, and MG conceived of the presented idea. All authors developed the theory and wrote parts of the manuscript, based on their respective expertise in the relevant concepts, i.e., (team) flow (CP and BKor), communication (BKoż and APo), management (OF and APy), and machine learning (MG, MN, and MI). CP supervised the writing process. All authors discussed the article and contributed to the final manuscript.

## Conflict of Interest

The authors declare that the research was conducted in the absence of any commercial or financial relationships that could be construed as a potential conflict of interest.

## Publisher’s Note

All claims expressed in this article are solely those of the authors and do not necessarily represent those of their affiliated organizations, or those of the publisher, the editors and the reviewers. Any product that may be evaluated in this article, or claim that may be made by its manufacturer, is not guaranteed or endorsed by the publisher.
